# Genetic diversity and differentiation among populations of the Indian eri silkworm, Samia cynthia ricini, revealed by ISSR markers

**DOI:** 10.1673/2006_6_30.1

**Published:** 2006-10-20

**Authors:** K. Vijayan, H. J. Anuradha, C. V. Nair, A. R. Pradeep, A. K. Awasthi, B. Saratchandra, S. A S. Rahman, K. C. Singh, R. Chakraborti, S. Raje Urs

**Affiliations:** 1 Seribiotech Research Laboratory, Central Silk Board, CSB Campus, Kodathi, Carmelram P.O., Bangalore 560 035, Karnataka, India; 2 Central Muga Eri Research and Training Institute, Jorhat, Assam, India

**Keywords:** Eri phenotype, geographic isolation, gene flow, heterozygosity

## Abstract

Samia cynthia ricini (Lepidoptera:Saturniidae), the Indian eri silkworm, contributes significantly to the production of commercial silk and is widely distributed in the Brahmaputra river valley in North-Eastern India. Due to over exploitation coupled with rapid deforestation, most of the natural populations of S. cynthia ricini are dwindling rapidly and its preservation has become an important goal. Assessment of the genetic structure of each population is a prerequisite for a sustainable conservation program. DNA fingerprinting to detect genetic variation has been used in different insect species not only between populations, but also between individuals within a population. Since, information on the genetic basis of phenotypic variability and genetic diversity within the S. cynthia ricini populations is scanty, inter simple sequence repeat (ISSR) system was used to assess genetic diversity and differentiation among six commercially exploited S. cynthia ricini populations. Twenty ISSR primers produced 87% of inter population variability among the six populations. Genetic distance was lowest between the populations Khanapara (E5) and Mendipathar (E6) (0.0654) and highest between Dhanubhanga (E4) and Titabar (E3) (0.3811). Within population, heterozygosity was higher in Borduar (E2) (0.1093) and lowest in Titabar (E3) (0.0510). Highest gene flow (0.9035) was between E5 and E6 and the lowest (0.2172) was between E3 and E5. Regression analysis showed positive correlation between genetic distance and geographic distance among the populations. The high G_ST_ value (0.657) among the populations combined with low gene flow contributes significantly to the genetic differentiation among the S. cynthia ricini populations. Based on genetic diversity, these populations can be considered as different ecotypes and *in situ* conservation of them is recommended.

## Introduction

Understanding and preserving biodiversity is one of the most important global challenges that biologists are facing. Widespread realization that biodiversity is strongly modified by changes in global environment has generated strategies to conserve and protect biodiversity in many parts of the world. Assessment of the genetic diversity present within a species is a prerequisite for developing a sustainable conservation program. North–eastern India belongs to the trans-Himalayan mountainous zone and is characterized by a tropical humid climate with temperature varying from 10 to 24° C, a range of relative humidity from 75–98% and rainfall of 1300–1700 mm per year. 65% of the area is mountainous with evergreen forests and it includes the Brahmaputra river valley. The forested areas are the abode of many valuable flora and fauna including about ten species of wild silk moths (Chowdhury 1983; Thangavelu 1991). The Indian eri silkworm, Samia cynthia ricini (Lepidoptera:Saturniidae), a commercial silk producing insect, is believed to have originated in the Brahmaputra valley (Jolly et al. 1979) and has had restricted distribution in India, China and Japan for two centuries (Peigler 1993; Singh and Benchamin 2002). The primary food plant of this polyphagous insect is castor (Ricinus communis L.), but it also feeds on a wide range of food plants such as Heteropanax fragrans Seem, Manihot utilissima Phol, Evodia flaxinifolia Hook, Ailenthus gradulosa Roxb etc (Suryanarayana et al. 2002). The wild S. cynthia ricini silkworm completes one to three generations per year depending on geographical position and climatic conditions of the region, however, up to six generations occur in the domesticated cultures (Neupane et al. 1990). Populations of S. cynthia ricini, that have been commercially exploited and are present in different regions of north-east India showed wide variations in morphological and quantitative characters such as absolute silk content, larval weight, cocoon weight, cocoon shell weight and silk ratio (Siddiqui et al. 2000). These populations were named as Nongpoh, Kokrajhar Red, Borduar, Titabar, Sille, Dhanubhanga, Mendipathar and Khanapara, after their place of collection from where they were originally reported and were abundantly present. Due to over exploitation of the silkworms for commercial uses coupled with deforestation, most of these natural populations are dwindling rapidly. In order to preserve the natural biodiversity present among these populations, attempts are being made to understand the genetic structure of each population. Preliminary studies based on some quantitative traits such as cocoon weight, shell weight, larval weight etc. were made to understand the genetic basis of this phenotypic variability (Siddiqui et al. 2000). However, no systematic studies were made to generate substantial information on the genetic diversity of these populations so as to develop appropriate strategy for its conservation at the natural habitat. The present study was focused on the genetic diversity of six commercial populations of S. cynthia ricini present in north-eastern India.

The advent of molecular biological techniques clearly showed the advantages of molecular markers over morphobiochemical markers to analyze population diversity. As the molecular markers are stable and environmentally independent, they are increasingly being preferred to phenotypic traits to detect genetic variation not only among populations but also between individuals within a population. A number of DNA marker systems such as simple sequence repeats (SSR; Kimpton et al. 1993; Estoup et al. 1993; Reddy et al. 1999a; Prasad et al. 2005), random amplified polymorphic DNA (RAPD; Williams et al. 1990; Nagaraja and Nagaraju 1995; Chatterjee and Pradeep 2003), inter-simple sequence repeats (ISSR; Zietkiewicz et al. 1994; Ehtesham et al. 1995; Reddy et al. 1999b; Chatterjee et al. 2004; Kar et al. 2005; Pradeep et al. 2005), expressed sequence tag (EST; Vlachou et al. 1997; Ciolfi et al. 2005) and amplified fragment length polymorphism (AFLP; Vos et al. 1995; Reineke et al. 1998;Katiyar et al. 2000) have been used to study the population genetics of different organisms including insects. Genetic diversity and differentiation among different populations of the wild silkworm Antheraea mylitta was examined using ISSR markers (Chatterjee et al. 2004; Kar et al. 2005). Use of SSR and EST primers requires prior knowledge of the genome of the organism whereas development of ISSR primers does not require prior information of the genome. The information available on the genome of S. cynthia ricini is inadequate to develop SSR and EST primers for assessing genetic diversity and genetic differentiation among its populations. As short repeats, such as dinucleotides, are ubiquitously distributed in eukaryotic genomes (Ichimura and Mita 1992; Goldstein and Schlötterer 1999; Prasad et al. 2005; Pradeep et al. unpublished), primers representing these repeats (eg. ISSR primers; Zietkiewicz et al. 1994) produce complex fingerprints from distantly related organisms. ISSR markers are reliable, reproducible, polymorphic and have been used to estimate genetic diversity among closely related populations also (Vogel and Scolnik 1997; Abbot 2001; Deshpande et al. 2001). Considering these advantages of ISSR primers, this marker system was used to analyze the genetic variability among S. cynthia ricini silkworm populations.

## Materials and Methods

### Genetic material and DNA extraction

**Figure 1:**
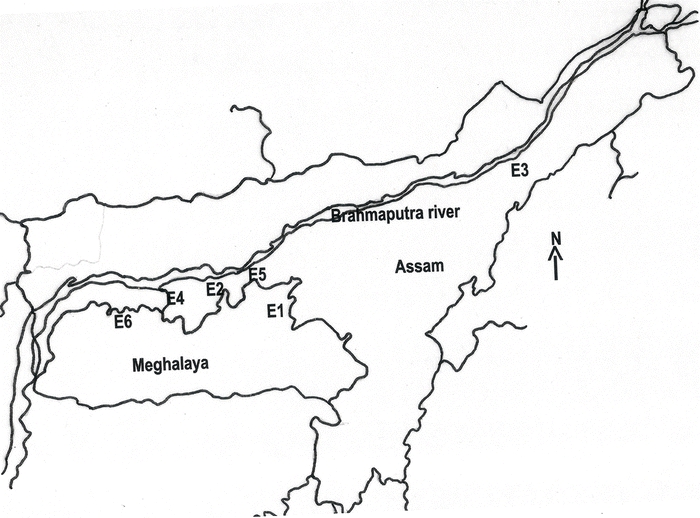
Map of North- Eastern India showing the places of collection of the eri silkworm, S.c. ricini populations. E1–E6 was the six populations as given in Table 1 (map not to scale).

Six morphologically distinct populations of S. cynthia ricini, collected from different regions of North-eastern India (Table 1, Fig. 1), were used for the study: Nongpoh (E1), Borduar (E2), Titabar (E3), Dhanubhanga (E4), Khanapara (E5) and Mendipathar (E6). From each population, 10 to 15 cocoons were collected and kept until emergence of the adult moth. Genomic DNA from 10 individual moths of each population was extracted separately following the phenol: chloroform extraction method (Suzuki et al. 1972). After RNAse incubation, DNA was re-extracted and the purified DNA was diluted in TE (Tris-EDTA; pH 8.0) buffer to obtain the concentration of DNA at 10ng/μl.

**Table 1:**
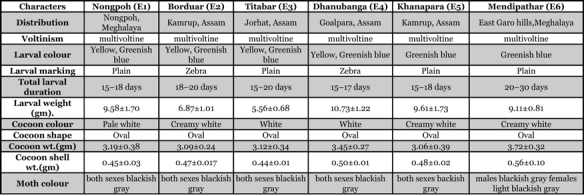
Key to the phenotypic characters of six different populations of the Indian eri silkworm, S.cynthia ricini

### PCR amplification of the genomic DNA with ISSR primers

ISSR primers (Table 2), procured from Dr. John B. Hobbs, Nucleic Acid & Protein Service, Biotechnology Laboratory, University of British Columbia, Vancouver, Canada, (http://naps.biotech.ubc.ca) (set # 9), were tested for their efficacy in amplification of DNA. Twenty primers that produced reproducible robust bands, which appeared consistently and distinctly across three different amplifications, were selected for the study. PCR amplification of the DNA was carried out as described earlier (Chatterjee et al 2004) on an MJ Research Thermal-Cycler, PTC 200 (MJ Research Inc. www.mjr.com/), using 20 μl reaction mixture containing 10x PCR buffer, 2 mM dNTPs, 2.5 mM MgCl_2_, 0.10 μl *Taq* DNA polymerase (recombinant) (5 U/μl) (all the chemicals from MBI Fermentas; Fermentas Inc, www.fermentas.com/), 2.0 μl of 1.5 μM ISSR primer and 40 ng DNA.

**Table 2:**
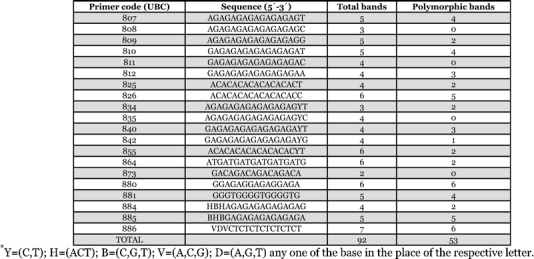
Polymorphism detected by 20 ISSR primers employed in the genetic diversity studies on six populations of eri silkworm Samia cynthia ricini

The PCR products were resolved on 1.5% agarose gel, in Tris - Boric Acid–EDTA buffer, stained with ethidium bromide (0.5 μg/ml) and photographed under Bioimaging system (Syngene, www.syngene.com). Binary scoring of the profiles was done on the basis of presence or absence of a band at a particular locus. The amplification of the DNA with each primer was repeated thrice and only the robust bands were scored for the analysis.

### Statistical analyses

From the binary data, genetic dissimilarity coefficients among the individuals were estimated using Dice’s coefficient (D) = 1–[(2N_ab_)/(2N_ab_ + N_a_ + N_b_)], where N_ab_ is the number of bands that are shared by the genotypes ‘a’ and ‘b’; N_a_ is the number of bands present in ‘a’ and N_b_ is the number of bands present in ‘b’ (Sneath and Sokal 1973). A phylogeny tree was generated from the above matrix using the unweighted pair group method with arithmetical averages (UPGMA) (Sneath and Sokal 1973). Bootstrap analysis was conducted using 10000 replicates as implemented in PAUP 4.0 software (Swofford 1999). Nei’s (1972) genetic distance among the phenotypes was calculated using POPGENE Version 1.3 (Yeh 1998). A dendrogram for the six populations was also created using UPGMA analysis (Sneath and Sokal 1973). The PhiPT value for genetic variability was calculated using GenAlEx v 5.1. The percentage of polymorphism, heterozygosity (h), number of alleles (Na), number of effective alleles (Ne) and Shannon’s information index (I) were calculated for each population. Genetic variability in the populations was also calculated using Nei’s (1973) coefficient of gene differentiation (G_ST_) in POPGENE version 1.3 (Yeh 1998). In POPGENE the genetic divergence among different populations was calculated using a multiallelic analogue of F_ST_ among a finite number of populations, which is otherwise, called the coefficient of gene differentiation (Nei 1973). This is stated as G_ST_= D_ST_/H_t_= (H_t_-H_s_) /H_t’_ where D_ST_ is the average gene diversity between subpopulations, including the comparisons of sub populations with themselves. The D_ST_= (H_t_-H_s_). G_ST_ is an extension of Nei’s (1972) genetic distance between a pair of populations to the case of hierarchical structure of populations (Nei 1973). H_t_ = (1–∑p_i_^2^), where p_i_ is the frequency of i^th^ allele at a locus in a population and ∑ is the summation of all alleles. Hence, the H_s_ in the equation were defined in terms of gene diversities. However, for random mating subpopulations, gene diversities can be defined as expected heterozygosities under Hardy-Weinberg equilibrium averaged among sub populations (H_s_) and of the total population (H_t_). The estimate of gene flow from G_ST_ was calculated as (Nm) = 0.5 (1 – G_ST_)/G_ST_. To analyze the relation between genetic distance and geographic distance between the populations, regression analysis was done using inter- population genetic distance against the geographic distance between them in kilometers. A scatter diagram was prepared and the best-fit linear regression line was applied.

## RESULTS

Six populations of S. cynthia ricini showed variation in morphological and quantitative characters (Table 1). The color of the mature larvae was greenish blue in E5 and E6, and a mixture of yellowish and greenish blue in E1, E2, E3 and E4. Typical zebra markings were present on larvae of E2 and E4 whereas E1, E3, E5 and E6 were plain white without any markings. The weight of mature larvae recorded was 9.58 ± 1.70 g in E1, 6.87 ± 1.01 g in E2, 5.56 ± 0.68 g in E3, 10.73 ± 1.22 g in E4, 9.61 ± 1.73 g in E5 and 9.11 ± 0.81 g in E6. Cocoon weight varied from 3.06 ± 0.39 g in E5 to 3.72 ± 0.32 g in E6.

### Inter population genetic diversity

**Figure 2:**
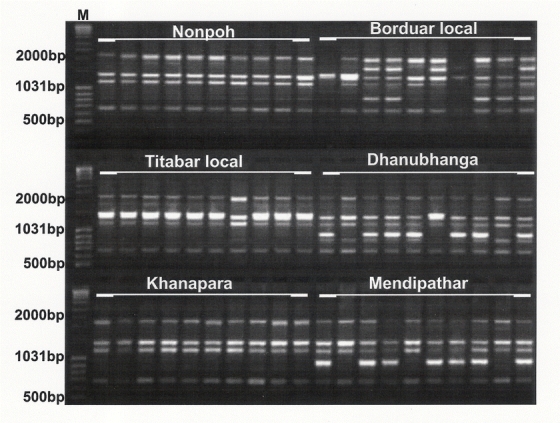
An example of PCR amplification profile generated from genomic DNA of S.c. ricini populations with UBC886, resolved on 1.5% agarose gel. M- marker. E1–E6 was the six populations as given in Table 1.

**Figure 3:**
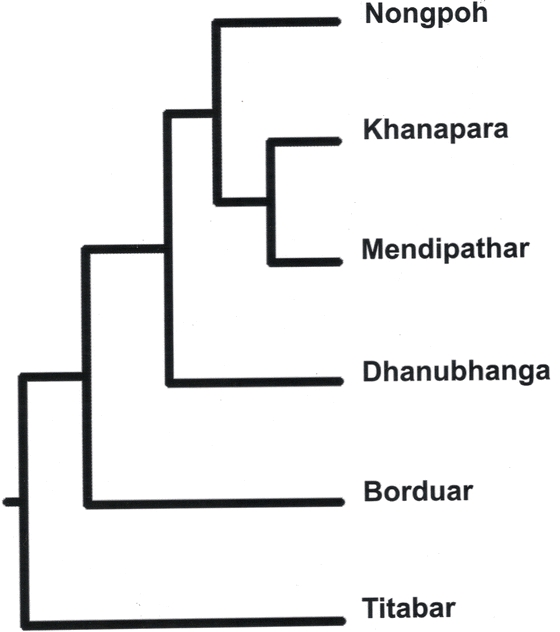
Dendrogram showing grouping of the six populations of S.c. ricini using UPGMA analysis based on Dice dissimilarity matrix. E1–E6 was the six populations as given in Table 1.

Twenty ISSR primers generated a total of 92 bands, of which 53 were polymorphic (Table 2; Fig.2). AMOVA revealed 87% of inter-population genetic variability and the PhiPT value was 0.872, which was significant at a probability of 0.010. Analysis for pair-wise genetic distance revealed that the genetic distance was minimum between E5 and E6 populations (0.0654) and maximum between E4 and E3 (0.3811) populations (Table 3). The intra- population variability in terms of DNA polymorphism was highest in E2 (31.52%) and least in E5 and E6 (14.13%) (Table.4). The dendrogram shown in Figure 3 that resulted from the genetic dissimilarity matrix using UPGMA (Sneath and Sokal 1973) revealed that E5 and E6 were genetically closer than the other populations, and E3 formed an isolate.

**Table 3:**
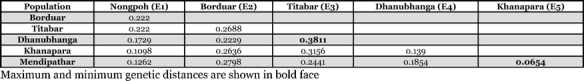
Genetic distance estimated among the six populations of eri silkworm, Samia cynthia ricini

### Intra population genetic diversity

**Figure 4:**
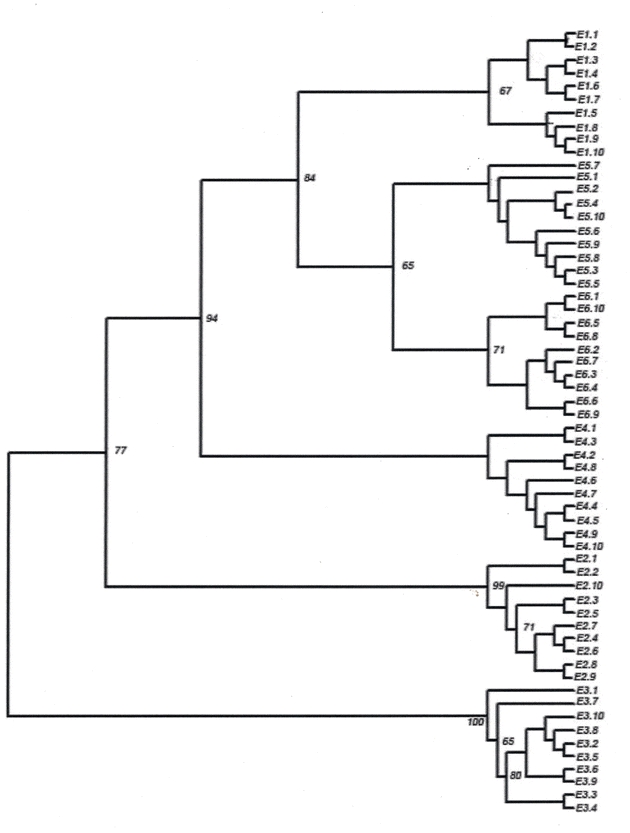
Dendrogram showing grouping of 60 individuals from six populations of S.c. ricini based on the genetic distance derived from ISSR markers. Bootstrap values were given at the fork of each group. E1–E6 was the six populations as given in Table 1. E1.1 – One individual from the population E1.

When all 60 individuals were taken for analysis without assigning any population status, the individuals grouped according to their population affinity (Fig. 4). None of the individuals changed its population cluster. The bootstrap values for each population were highly significant and varied from 65 to 100. The grouping of individuals within each population showed variation. Though the individuals of different populations grouped into sub groups, in most cases, the bootstrap values were insignificant. But significantly high bootstrap values (71–99%) were observed in the subgrouping of the E2 population.

### Population genetic parameters

The population diversity analysis using POPGENE revealed that the average number of observed alleles varied from 1.143 in E5 and E6 to 1.315 in E2. The heterozygosity present within a population was higher in E2 (0.109) and was lowest in E3 (0.051) (Table 4). When all populations were considered, the total genetic diversity (Ht) was 0.233. The total genetic differentiation coefficient (G_ST_) among the populations was 0.657 (Table 5). The pair-wise comparison of the populations showed that the genetic differentiation varied from 0.356 between E5 and E6 to 0.697 between E3 and E5. Gene flow among the populations was 0.261. Pair-wise analysis of the populations for their genetic differentiation and gene flow showed that the highest gene flow (0.904) was between E5 and E6 and lowest (0.217) was between E3 and E5 (Table 5). E3 showed an average gene flow estimate of 0.257 with all other populations.

**Table 4:**
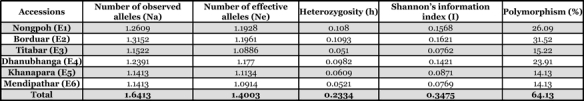
Gene diversity in the six populations of the eri silkworm Samia cynthia ricini

**Table 5:**
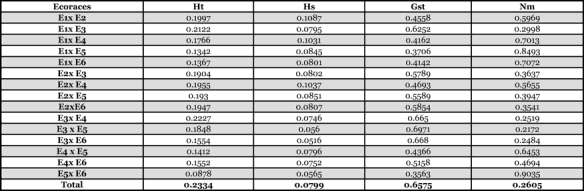
Pair-wise estimation of genetic differentiation and gene flow among six populations of Samia cynthia ricini

### Genetic diversity and geographical distribution

**Figure 5:**
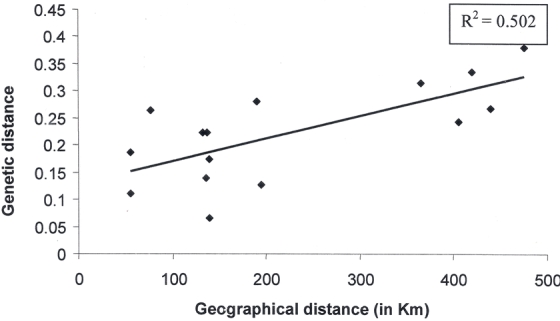
Regression of genetic distance between the populations of eri silkworm, S.c. ricini against geographical distance between their places of collection.

In north-eastern India, S. cynthia ricini was distributed in the Brahmaputra river valley of Assam and in the hilly area. Titabar, where the population E3 was collected from, is located in the Upper Assam area and is approximately 421 kilo meters away from all other places of collection. The distance between the other populations was comparatively smaller. Regression of the genetic distance between the populations against geographic distance between them showed a positive correlation (R^2^ = 0.502) (Fig. 5).

## DISCUSSION

Recent advances in silkworm genome analysis showed the presence of large numbers of repeats in the domesticated mulberry silkworm Bombyx mori (Mita et al. 2004; Biology Analysis group et al. 2004; Prasad et al. 2005). S. cynthia ricini seemed to share about 13% of its microsatellites with B. mori (Prasad et al. 2005). The presence of 250 mariner-like elements was reported in Philosamia cynthia ricini and were found to be similar to the *Mos*I element from Drosophila mauritiana (Prasad et al. 2002). Genetic divergence among closely related populations of B. mori and in A. mylitta was successfully deciphered using ISSR profiling (Chatterjee et al. 2004; Pradeep et al. 2005;Kar et al. 2005). These studies explicitly report the importance of repeats and transposable elements in the molecular phylogenetic analysis of domesticated and wild silkworms. The higher amount of genetic divergence realized in this study from closely related populations of S. cynthia ricini, further demonstrates the efficacy of the ISSR marker system. The ISSR markers revealed different levels of genetic differentiation among the populations of S. cynthia ricini. However, when a dominant marker system such as ISSR is used and the PCR products are resolved on agarose gels, a homozygote for a null allele at a particular locus will not produce a band, but both a heterozygote and a homozygote at that locus will produce a band. As a result, the fundamental data available from a population at any given locus are the number of individuals with a band corresponding to that locus and the number of individuals lacking that band. Nevertheless, ISSR markers showed the potential not only to reveal variations among geographically separated populations, but also among individuals within populations.

The genetic diversity revealed in this study is of much use in formulating strategies to conserve the genetic diversity present in this unique silkworm species. The low genetic variation revealed by AMOVA (ca. 13%) within the individuals of a population points to the fact that the populations are homogenous in nature, whereas the higher genetic variability (ca. 87%) among the populations indicated that the populations have already differentiated into separate genetic pools. Hence, these different gene pools should be conserved separately and maintained without any inter-mixing. Comparatively higher polymorphism (31.52%), heterozygosity (0.1093) and high boots trap values (98%) at the subgroup level observed in the E2 population points to significantly higher genetic differentiation at Borduar. Since moths of S. cynthia ricini can fly only a maximum of two kilometers, the chances of natural genetic mixing between the geographically separated populations can be ruled out. Transportation of populations for multiplication and unscientific breeding between S. cynthia ricini populations leads to genetic mixing of the populations. Such human interventions should be avoided at the core germplasm level to maintain purity of the germplasm.

The high gene flow (0.9035) between the populations E5 and E6 is quite understandable as they are similar in phenotypic traits, such as cocoon color (greenish blue). The high phenotypic and genetic similarity and gene flow observed within these two populations suggests its common origin and later succession into different populations by adapting to the varying climatic conditions. Since the E3 (Titabar) population is genetically and geographically distanced from all other populations and seems to be an isolated one, detailed molecular analysis of this population is required to decipher its origin. The E3 population exhibited comparatively higher G_ST_ (0.657) combined with low gene flow (Nm = 0.2762) that has contributed significantly to genetic differentiation between populations (Wright 1978; Hartl and Clark 1997). The genetic distance of E3 from all other populations was found to be positively correlated with the geographic distance between Titabar and the natural habitats of other populations. This confirms an independent and slow isolated genetic differentiation of E3 at the high altitude of upper Assam where within population heterozygosity and gene flow was found to be low, and the possibility of genetic mixing seemed negligible. The independent genetic differentiation of this population is attributed to the strong effect of isolation by geographical distance. In fact, geographical isolation, mutation and selection are the most likely forces of population differentiation. This kind of association between geographic components and genetic differentiation was observed in natural populations of Drosophila (Frydenberg et al. 2003; Anderson et al. 2005). S. cynthia ricini silkmoths are poor flyers with a short life span, which is likely to reduce chances of genetic mixing among geographically close populations. This could account for the genetic differentiation among the geographically close S. cynthia ricini populations of Lower Assam. It is a known fact that high gene flow between populations precludes local adaptation that results from fixation of alleles favoured by local climatic conditions, and will prevent speciation (Barton and Hewitt 1985). At the same time gene flow generates new polymorphisms and new gene combinations in populations, upon which selection can act.

Thus from this study it is clear that the populations evaluated not only differ in their phenotypic traits but also in their genetic make up. Since S. cynthia ricini is believed to have originated in the Brahmaputra river valley of north-eastern India and this is the region where maximum genetic diversity is expected, the populations identified as genetically divergent have to be conserved *in situ*. The high economic value of the silk industry further emphasizes the need for urgent measures in this direction.

## Abbreviations

Samia cynthia ricini populations: Nongpoh (E1), Borduar (E2), Titabar (E3), Dhanubhanga (E4), Khanapara (E5), Mendipathar (E6)

## References

[i1536-2442-6-30-1-Abbot1] Abbot P. 2001 Individual and population variation in invertebrates revealed by inter-simple sequence repeats (ISSRs) Journal of Insect Science 1 8 http://insectscience.org/1.8 PMC355892 15455068

[i1536-2442-6-30-1-Anderson1] Anderson A. R. Hoffmann A. A. McKechnie S. W. Umina P. A. Weeks A. R. 2005 The latitudinal cline the in (3R) Payne inversion polymorphism has shifted in the last 20 years in Australian Drosophila melanogaster populations Molecular Ecology 14 851 858 15723676 10.1111/j.1365-294X.2005.02445.x

[i1536-2442-6-30-1-Barton1] Barton N. H. Hewitt G. M. 1985 Analysis of hybrid zones Annual Review of Ecology and Systematics 16 113 148

[i1536-2442-6-30-1-Analysis1] Biology analysis group 2004 A draft Sequence for the genome of the domesticated silkworm (Bombyx mori) Science 306 1937 1940 15591204 10.1126/science.1102210

[i1536-2442-6-30-1-Chatterjee1] Chatterjee S. N. Pradeep A. R. 2003 Molecular markers (RAPD) associated with growth, yield and origin of the silkworm, Bombyx mori in India Russian Journal of Genetics 39 1612 1624 14964827

[i1536-2442-6-30-1-Chatterjee2] Chatterjee S. N. Vijayan K. Roy G. C. Nair C. V. 2004 ISSR profiling of genetic variability in the ecotypes of Anthereae mylitta Drury, the tropical tasar silkworm Russian Journal of Genetics 40 152 159 15065428

[i1536-2442-6-30-1-Chowdhury1] Chowdhury S. N. 1983 Eri silk Industry Directorate of Sericulture & Weaving, Government of Assam India

[i1536-2442-6-30-1-Ciolfi1] Ciolfi S. de Filippis T. Torti C. Malacrida A. R. Dallai R. 2005 Molecular characterization and chromosomal localization of female-specific genes from the Mediterranean fruit fly Ceratitis capitata (Diptera: Tephritidae) Genome 48 139 144 15729405 10.1139/g04-080

[i1536-2442-6-30-1-Deshpande1] Deshpande K. U. Apte G. S. Bahulikar R. A. Lagu M. D. Kulkarni B. O. Suresh H. S. Singh N. P. Rao M. K. Gupta V. S. Pant A. Renjekar P. K. 2001 Genetic diversity across natural populations of montane plant species from the Western Ghats, India revealed by inter-simple sequence repeats Molecular Ecology 10 2397 2408 11742544 10.1046/j.0962-1083.2001.01379.x

[i1536-2442-6-30-1-Estoup1] Estoup A. Solignac M. Harry M. Cornuet J. M. 1993 Characterization of (GT)_n_ and (CT)_n_ microsatellites in two insect species: Apis mellifera and Bombus terrestris. Nucleic Acids Research 21 1427 1431 8464734 10.1093/nar/21.6.1427 PMC309328

[i1536-2442-6-30-1-Ehtesham1] Ehtesham N. Z. Bentur J. S. Bennett J. 1995 Characterization of a DNA sequence that detects repetitive DNA elements in the Asian gall midge (Orseolia oryzae) genome: Potential use in DNA fingerprinting of biotypes Gene 153 179 183 7875586 10.1016/0378-1119(94)00769-o

[i1536-2442-6-30-1-Frydenberg1] Frydenberg J. Hoffmann A. A. Loeschcke V. 2003 DNA sequence variation and latitudinal associations in hsp23, hsp26 and hsp27 from natural populations of Drosophila melanogaster. Molecular Ecology 12 2025 32 12859626 10.1046/j.1365-294x.2002.01882.x

[i1536-2442-6-30-1-Goldstein1] Goldstein D. B. Schlötterer C. 1999 Microsatellites: Evolution and Applications Oxford University Press Oxford

[i1536-2442-6-30-1-Hartl1] Hartl D. L. Clark A. G. 1997 Principles of population genetics, 3^rd^ edition Sinauer Associates, Inc

[i1536-2442-6-30-1-Ichimura1] Ichimura S. Mita K. 1992 Essential role of duplications of short motif sequences in the genomic evolution of Bombyx mori. Journal of Molecular Evolution 35 123 130 1501252 10.1007/BF00183223

[i1536-2442-6-30-1-Jolly1] Jolly M. S. Sen S. K. Sonwalker T. N. Prasad G. K. 1979 Non-mulberry silks In: Manual on Sericulture, G Rangaswami, MN, Narasimhanna, K, Kashivishwanathan, CR, Sastri, MS Jolly, editors 1 178 Food and Agriculture Organization of the United Nations Rome

[i1536-2442-6-30-1-Kar1] Kar P. K. Vijayan K. Nair C. V. Mohandas T. P. Saratchandra B. Thangavelu K. 2005 Genetic variability and genetic structure of wild and semi-domestic populations of tasar silkworm (Antheraea mylitta ) ecorace Daba as revealed through ISSR markers Genetica 125 173 183 16247690 10.1007/s10709-005-7002-z

[i1536-2442-6-30-1-Katiyar1] Katiyar S. K. Chandel G. Tan Y. Zhang Y. Huang B. Nugaliyaddar L. Fernando K. Bentur J. S. Inthavong S. Constantino S. Bennett J. 2000 Biodiversity of Asian rice gall midge (Orseolia oryzae Wood Mason) from five countries examined by AFLP analysis Genome 43 322 332 10791821

[i1536-2442-6-30-1-Kimpton1] Kimpton C. P. Gill P. Walton A. Urquhart A. Millican E. S. Adams M. 1993 Automated DNA profiling employing multiplex amplification of short tandem repeat loci PCR methods and applications 3 13 22 8220182 10.1101/gr.3.1.13

[i1536-2442-6-30-1-Mita1] Mita K. Kasahara M. Sasaki S. Nagayasu Y. Yamada T. 2004 The genome sequence of silkworm, Bombyx mori DNA Research 11 27 35 15141943 10.1093/dnares/11.1.27

[i1536-2442-6-30-1-Nagaraja1] Nagaraja G. M. Nagaraju J. 1995 Genome fingerprinting in silkworm, Bombyx mori, using random arbitrary primers Electrophoresis 16 1633 1638 8582347 10.1002/elps.11501601270

[i1536-2442-6-30-1-Nei1] Nei M. 1972 Genetic distance between populations American Naturalist 106 283 292

[i1536-2442-6-30-1-Nei2] Nei M. 1973 Analysis of gene diversity in subdivided populations Proceedings of National Academy of Sciences, USA 70 3321 3323 10.1073/pnas.70.12.3321 PMC427228 4519626

[i1536-2442-6-30-1-Neupane1] Neupane F. P. Thapa R. B. Parajulee M. N. 1990 Life and seasonal histories of the eri silkworm, Samia cynthia ricini Hutt. (Lepidoptera: Saturniidae), in Chitwan, Nepal Journal of Institute of Agriculture: Animal Science 11 113 120

[i1536-2442-6-30-1-Peigler1] Peigler R. S. 1993 Wild silks of the world American Entomologist 39 151 161

[i1536-2442-6-30-1-Pradeep1] Pradeep A. R. Chatterjee S. N. Nair C. V. 2005 Genetic differentiation induced by selection in an inbred population of the silkworm Bombyx mori, revealed by RAPD and ISSR marker systems Journal of Applied Genetics 46 291 298 16110186

[i1536-2442-6-30-1-Prasad1] Prasad M. D. Nurminsky D. L. Nagaraju J. 2002 Characterization and molecular phylogenetic analysis of mariner elements from wild and domesticated species of silkmoths Molecular Phylogenetics and Evolution 25 210 217 12383762 10.1016/s1055-7903(02)00225-7

[i1536-2442-6-30-1-Prasad2] Prasad M. D. Muthulakshmi M. Madhu M. Archak S. Mita K. Nagaraju J. 2005 Survey and analysis of microsatellites in the silkworm, Bombyx mori: Frequency, distribution, mutation, marker potential and their conservation in heterologous species Genetics 169 197 214 15371363 10.1534/genetics.104.031005 PMC1448858

[i1536-2442-6-30-1-Reddy1] Reddy K. D. Nagaraju J. Abraham E. G. 1999a Genetic characterization of silkworm Bombyx mori by simple sequence repeats (SSR) – anchored PCR Heredity 83 681 687 10651912 10.1046/j.1365-2540.1999.00607.x

[i1536-2442-6-30-1-Reddy2] Reddy K. D. Abraham E. G. Nagaraju J. 1999b Microsatellites of the silkworm, Bombyx mori: abundance, polymorphism and strain characterization Genome 42 1057 1065 10659770

[i1536-2442-6-30-1-Reineke1] Reineke A. Karlovsky P. Zebitz C. P. W. 1998 Preparation and purification of DNA from insects for AFLP analysis Insect Molecular Biology 7 95 99 9459433 10.1046/j.1365-2583.1998.71048.x

[i1536-2442-6-30-1-Siddiqui1] Siddiqui A. A. Saha L. M. Das P. K. 2000 Genetic variability and correlation studies of some quantitative traits in Eri silkworm International Journal of wild silkmoth and silk 5 234 237

[i1536-2442-6-30-1-Singh1] Singh K. C. Benchamin K. V. 2002 Biology and ecology of the eri silkmoth Samia ricini (Donovan) (Saturniidae): a review Bulletin of Indian Academy of Sericulture 6 20 33

[i1536-2442-6-30-1-Sneath1] Sneath P. H. Sokal R. R. 1973 Numerical taxonomy W. H. Freeman San Francisco

[i1536-2442-6-30-1-Suryanarayana1] Suryanarayana N. Sarmah M. C. Sahu A. K. Kumar A. Das P. K. 2002 Status paper on promotion of eri culture in India Proceedings of National Workshop on Sericulture Germplasm Management & Utilization 50 53 CSGRC Hosur, India

[i1536-2442-6-30-1-Suzuki1] Suzuki Y. Gage L. Brown D. D. 1972 The genes for silk fibroin in Bombyx mori. Journal of Molecular Biology 70 637 649 5083150 10.1016/0022-2836(72)90563-3

[i1536-2442-6-30-1-Swofford1] Swofford D. L. 1998 PAUP*: Phylogenetic analysis using parsimony (*and other Methods), Version 4 Sinauer Associates Sunderland, MA

[i1536-2442-6-30-1-Thangavelu1] Thangavelu K. 1991 Wild sericigenous insects of India: A need for conservation In: Wild Silkmoths’91 71 77 International Society for wild silkmoths Japan

[i1536-2442-6-30-1-Vlachou1] Vlachou D. Konsolaki M. Tolias P. P. Kafatos F. C. Komitopoulou K. 1997 The autosomal chorion locus of the medfly, Ceratitis capitata. I. Conserved synteny, amplification and tissue specificity but sequence divergence and altered temporal regulation Genetics 147 1829 1842 9409839 10.1093/genetics/147.4.1829 PMC1208349

[i1536-2442-6-30-1-Vogel1] Vogel J. M. Scolnik P. A. 1997 Direct amplification from microsatellites: detection of simple sequence repeat-based polymorphisms without cloning In: Caetano-Anolles G, Gresshoff PM, editors. DNA markers Protocols, Applications, and Overviews 133 150 Wiley-VCH New York

[i1536-2442-6-30-1-Vos1] Vos P. Hogers R. Bleeker M. Reijans M. van de Lee T. Hornes M. Frijters A. Pot J. Peleman J. Kuiper M. Zabeau M. 1995 AFLP: a new technique for DNA fingerprinting Nucleic Acids Research 23 4407 4414 7501463 10.1093/nar/23.21.4407 PMC307397

[i1536-2442-6-30-1-Wright1] Wright S. 1978 Evolution and Genetics of population variability within and among natural populations The University of Chicago Press Chicago

[i1536-2442-6-30-1-Williams1] Williams J. G. K. Kubelik A. R. Livak K. J. Rafalski J. A. Tingey S. V. 1990 DNA polymorphisms amplified by arbitrary primers are useful as genetic markers Nucleic Acids Research 18 6531 6535 1979162 10.1093/nar/18.22.6531 PMC332606

[i1536-2442-6-30-1-Yeh1] Yeh F. C. 1998 POPGENE16. Version 1.31 http://www.ualberta.ca/~fyeh Agriculture and Forestry Molecular Biology and Biotechnology center, University of Alberta and Center for International Forestry Research Canada

[i1536-2442-6-30-1-Zietkiewicz1] Zietkiewicz E. Rafalski A. Labuda D. 1994 Genome fingerprinting by simple sequence repeat (SSR) - anchored polymerase chain reaction amplification Genomics 20 176 183 8020964 10.1006/geno.1994.1151

